# An evolutionary approach to Function

**DOI:** 10.1186/2041-1480-1-S1-S4

**Published:** 2010-06-22

**Authors:** Phillip Lord

**Affiliations:** 1 School of Computing Science, Newcastle University, Newcastle-Upon-Tyne, NE1 7RU, UK

## Abstract

**Background:**

Understanding the distinction between function and role is vexing and difficult. While it appears to be useful, in practice this distinction is hard to apply, particularly within biology.

**Results:**

I take an evolutionary approach, considering a series of examples, to develop and generate definitions for these concepts. I test them in practice against the Ontology for Biomedical Investigations (OBI). Finally, I give an axiomatisation and discuss methods for applying these definitions in practice.

**Conclusions:**

The definitions in this paper are applicable, formalizing current practice. As such, they make a significant contribution to the use of these concepts within biomedical ontologies.

## Background

Large parts of modern biology are aimed at answering questions about function. For example, much of the Gene Ontology deals with molecular function [1]. In dealing with the social aspects of science, roles are similarly important. It is clear, therefore, that function and role are important concepts in biomedical ontologies and are prime candidates for inclusion in an upper ontology. A coherent, consistent and shared definition for function and role is likely to decrease the effort required to integrate independently-developed ontologies.

One upper ontology, the Basic Formal Ontology (BFO) [2], currently in use by a number of groups, includes a definition of function. However, this definition is not naturally applicable to biology; it is not clear, for instance, that Gene Ontology molecular functions are also BFO functions, as discussed later (see ''Biological Function''). An alternative is available within the General Formal Ontology (GFO), which provides an ontology of function [3]. This ontology provides a more extensive framework for describing functions but, in itself, does not define biological function. Despite this, there is a reasonable degree of informal agreement among biologists as to the meaning of the word. Conversely, while formal and informal definitions for role seems clear, many people have difficulties in applying it in practice.

In this paper, I address two key issues relating to the modeling of function and role coherently for biomedical ontologies: firstly, how I unify the definitions of function as they apply to artifacts and to life; and, secondly, how do I differentiate between roles and functions? I consider illustrative examples where the answers are reasonably clear and evolve a definition from them. I then consider examples of the application of current definitions from BFO in a practical biomedical use. Finally, I offer an axiomatisation in OWL stemming from my definitions and consider how they could be applied in practice.

## Results

### Biological Function

First, consider the current definition of function provided by BFO (see Def: 1).

**Definition 1*** Function is a realizable entity the manifestation of which is an essentially end-directed activity of a continuant entity being a specific kind of entity in the kind or kinds of context that it is made for*

As a simple example, a hammer (*the continuant entity*) was made to hammer nails (*the function*) in a hammering process (*the end-directed activity*). This definition is problematic for biological systems. The problem here is simple: most biological systems were not made or designed for any purpose. Although, it has not been incorporated into BFO yet, there is a potential definition for 'biological function" which would become a new child of function (Def: 2) [4].

**Definition 2*** A biological function is a function which inheres in an independent continuant that is i) part of an organism and ii) exists and has the physical structure as a result of the coordinated expression of that organism's structural genes.*

As an example, a foot (*the independent continuant*) is part of an organism, exists, and develops in a controlled way as a result of gene expression. There are some difficulties with this definition. Consider the following examples: a differentiated tumour has its structure through coordinated expression of its genes, it exists and it engages in end-directed activity; a male ant has its structure as a result of gene expression, and engages in end-directed activity, however, it is not part of an organism. From this, I conclude that a differentiated tumour does, indeed, have a function (growing). A male ant, however does not have a function. Also consider molecular function: the physical structure of a protein is independent of the expression of an organisms structural genes – only its presence depends on this. A protein, therefore, does not have a function, by this definition. So, there are two key problems: the definition does not work for entities above or below a certain size; and most biological entities have their structure as a result of coordinated expression. I offer the following alternate definition (Def: 3).

**Definition 3*** A biological function is a realizable entity that inheres in a continuant which is realized in an activity, and where the homologous structure(s) of individuals of closely related and the same species bear this same biological function.*

The definition given uses the notion of homology; evolution is key to our understanding of biology and it is appropriate that it should be used to define biological function. If a biological structure has a function, then this function will have evolved along with the structure; so, other structures with a common evolutionary descent will display the same behaviour. This definition also mirrors closely normal biological practice; the most common way to determine the function of an unknown structure is to look for function of a homologous structure.

It should be noted that this definition of biological function is* not* circular, although it has itself as part of its definition; rather it is recursive; a chimp hand and a human hand can have the same function because of each other. It does require that a structure must have a homolog for it to have a function. It does not require that these homologous structures be extant.

Applying this to our examples: the tumour now has no function because it has no homologs (different tumours arise as independent events and share no common ancestor). Likewise, the activity of the male ant now clearly is a function, as many different, related organisms behave in a similar way. Finally, a protein may have a function depending on the activity of its homologs. In short, this definition results in the same conclusions as our biological understanding.

### Relating the Functions

As well as biological function, another subclass,* artifactual function* has been suggested [4]. Next, I consider the relationship between function and these biological and artifactual subclasses. Taking an illustrative example, consider the sole of my foot and the sole of my shoe. They appear to operate to: provide a frictional surface to enable motion; provide padding to* reduce shock* to everything above; be tough enough to resist abrasion. They would appear to have the same function; indeed, like many artifacts, we would guess that the shoe owes much of its design to mimicry of biology. It would seem, therefore, sensible that instances of* Shock Resistance* could be either a biological or artifactual function. The alternative would be to duplicate many functions under both subclasses ("biological shock resistance", and "artifactual shock resistance") with very similar definitions. Some functions such as reproduction have to be a biological function, while others liquifying iron can never be. In short, whether an instance of function is biological or artifactual should be determined from the nature of the entity in which it inheres, rather than the process by which it is realized. I therefore offer a simple definition of function which reflects this (Def: 4).

**Definition 4*** A function is a realizable entity which is a biological function or an artifactual function.*

For the purposes of this paper, I note that the definition given for function earlier (Def: 1), can serve as a reasonable definition for artifactual function.

It is also interesting that this definition covers some unusual but non-pathological examples. Take a bacterium whose colonies change colour depending on the presence of a toxin and which was produced using synthetic biology techniques. The components have all evolved, but the organisation has not. Is the detection of the toxin then a biological or artifactual function? This is clearly a difficult, if uninspiring, question but given Def: 4, one that can avoid by simply describing it as a function; perhaps more intuitively, it can be described as both a biological and an artifactual function, suggesting strongly that these two classes should not be disjoint.

### Roles

Next, having considered the definition of function and its applicability to biology, I consider the issue of roles. The current definition (Def: 5) is complex; put more simply, it suggests that the entity having that role can be involved in an activity but that it was not necessarily intended for, nor necessarily has the structure for this.

**Definition 5*** A realizable entity the manifestation of which brings about some result or end that is not essential to a continuant in virtue of the kind of thing that it is but that can be served or participated in by that kind of continuant in some kinds of natural, social or institutional contexts.*

Consider the relationship between role and function. Again, I shall use a simple biological example, in this case of a man walking on his hands. By our earlier definition (Def: 3), "to walk on hands'' is* not* a function. While the homologous structure is, indeed, used for walking on in all closely related species, most humans do not walk on their hands. It would, therefore appear to be a role. In this context the hand has a role of* Shock Resistance.* This realizable entity also appears in the hands of many other primates; in this case, however, it would appear to be a function of the primate hand, as it is a function of their feet. I am left with a similar conclusion as previously. Just as Shock Resistance maybe either a biological or artifactual function, I must also conclude that the individuals of the same class can be a role, depending on the nature of the relationship between the independent continuant and realizable entity.

### OBI as a case study

So far in this paper, I have considered a number of illustrative examples and used these to draw conclusions about definitions for functions and roles. This methodology is appropriate, but has the limitation that the choice of other examples may have led us to different conclusions. In this section, therefore, I will consider the use that OBI (Ontology for Biomedical Investigations) [5] has made of function and role (Analysis was performed on OBI rc-1, (release 2009-11-06)). I choose to use OBI as it was built after BFO and with knowledge of it; many of the ontologies available from OBO were started without its use or knowledge. Considering first the functions of which OBI has 38. Can these functions be fulfilled by an entity which was not designed for the purpose? As shown in Table 1, most of them can, often by considering a highly generic device (like a computer) or organism (like a human). Some are highly generic in themselves and can be performed by many things (heat for example). I find no cases where a function could not be fulfilled by an entity which was not designed for the purpose. I consider "Molecular Function" to be out-of-scope for this section as it is not clear whether it fulfils the BFO definition of function.

**Table 1:** OBI Functions considered as Roles.

Function Bearer	OBI Function
Human	Perturb, Measure, Separation (3), Sort
Computer	Information Processor (3), Consume Data
Highly Generic	Freeze, Heat, Environment Control, Mechanical, Record, Contain, Transfer, Cool, Connection, Synthesizing, Excitation (2), Ionization, Energy Supply (1)
Distant Galaxy	Magnify
Out of Scope	Molecular Function (3)

I provide suggestions for entities that could engage in the same end-directed activity, but without being designed for the purpose. "Function" has been omitted from the OBI term names. Numbers indicate child terms which have been omitted for brevity. I do not consider "Information Processor" to be the function of a computer, as the definition is more specific than the term suggests.

Next, I consider OBI roles. There are many more roles than functions – around 90 in fact. Due to the size, here I consider whole branches of the role hierarchy.

There are a set of roles relating to reagents and their states. While label role (defined as a reagent role realized in a detection of label assay) seems sensible, it would appear that S^35^ CTP, used to label DNA, is manufactured specifically for this purpose. It certainly does not occur in nature. It would appear to fulfil the requirements for a function.

Next, let us consider reference substance roles (a role which can support the observation of similarities, differences, relative magnitude or change). In many cases, biological assays use a reference which is not manufactured. However, consider λ-HindIII fragments or a calibration standard. These would all appear to function as an reference and have been produced specifically for this purpose.

Finally, there are a number of roles for molecules or organisms: antigen role, pathogen role and primer role. In an age where we can engineer the production of DNA, protein and organisms, it is not clear that these can only ever be roles and not functions.

As a result of this analysis, I suggest a modification to the current definition of role (Def: 5). Both roles and functions can become apparent (*realized*) in natural, social or institutional contexts. That such a context exists does not provide a clear differentation between role and function; the critical distinction relates to whether the entity in question was designed to be or has homologs that are engaged in a given process. I suggest, therefore, this alternate and simpler definition for role (Def: 6).

**Definition 6*** A realizable entity the manifestation of which brings about some result or end that is not essential to a continuant because of its kind.*

In short, from this case study, I conclude that the role/function distinction is not clear. While OBI has a specific intent in mind with its application of the distinction (broadly and not exhaustively, social or experimental roles, device or instrument functions), this distinction is not the distinction made in the current definitions of role and function in BFO; further given that most functions could also appear to be roles, and many roles appear also to be functions, I suggest that the distinction made in the current definitions is not useful in the context of OBI.

The earlier theoretical analysis seems to be confirmed in practice within OBI. This suggests that the limitations in the definitions drawn from the earlier illustrative examples are general and not simply as a result of the specific examples chosen.

### An axiomatisation for function and role

I have produced an axiomatisation in OWL of functions and roles as defined in this paper, available as described in the abstract; due to space considerations I report here key differences between this and the BFO axiomatisation.

• There is an explicit relationship between RealizableEntity and Process. Subclasses use a more specific relationship. So, a ToAbsorbShock function may only be realized by a AbsorbtionOfShock process, if it is realized at all.

• Function and Role are defined classes. Stating that an instance of ToAbsorbShock is_function_of instance of FootSole implies, therefore, that the former is a function.

• Most leaves of RealizableEntity are direct children of RealizableEntity, with a few exceptions (ToReproduce is a child of BiologicalFunction).

The definitions could be extended further; for simplicity, I have not added classes to differentiate between organisms and artifacts. These could be added to automate the population of BiologicalFunction and ArtifactualFunction.

At the current time, it remains an open question whether Role, Function and its children should be disjoint. The key example of the function of a synthetic biological organism suggests that Biological and ArtifactualFunction function should not be disjoint (as it appears to be both), but I have no example which suggests whether Role and Function should be disjoint. In axiomatisating the examples given, these disjoint statements make no practical difference.

Many ontologies are built using the OBO format; while this has a slightly weaker semantics than OWL it is possible to represent much of OWL in OBO format [6]. The axiomatisation presented here can be represented using OBO* union* and* intersection of* to describe classes, which is usually translated as a definition. The universal link between RealizableEntity and Process has no natural equivalent. However, as this link has its own specific relationship which is restricted to this use, problems caused by the lack of an inexact semantic equivalent are likely to be relatively minor.

The axiomatisation presented here is related to that produced by others; Dumontier [7] focuses more on roles, while Burek et al. [3] provides for a more complex representation, covering issues such as preconditions.

### Applying the Definitions in Practice

Finally, I consider whether these definitions are* applicable*; for a given set of entities how do we decide whether we have a function (of either subclass) or a role.

The definition of an artifactual function easily allows its application: first, we determine whether the entity in question is an organism or part of one (which it should not be); second, we could ask whoever produced the entity what it was designed for. Of course, the second may not always be possible, in which case, we can guess from its design what its purpose is. In most cases, these questions will provide a clear answer. For biological function, the situation is less clear. Whether an entity is an organism or part of one is, in practice, likely to be straightforward for extant entities; otherwise, we can apply palentological techniques. Likewise, identification of closely related species and homologous structures is well known as it forms the basis of taxonomy. While developing an exact definition for "closely related" is outside the scope of this paper, it is possible.

The definition that I introduce for Function in this paper (Defn 4) is conjunctive; it is either biological or artifactual. Here I have given little evidence that these are the only kind of function. Fundamentally, these two arise from very different mechanisms. There could be other appropriate subclasses of function; the most obvious possibility would be Chemical Function. However, artifacts are designed by humans who understand, mimic and improve on biology by building tools. It is this mimicry that we wish to reflect with a common definition joining biological and artifactual function; this is not true for Chemical Function. To determine whether something is a role, it is possible to make a determination on the basis of whether the context is optional [4]. However, this optionality is a difficult criterion; firstly, all RealizableEntity's are optional in the sense that they might never be realized and, secondly, the optionality can depend on how specifically we define the bearer. A hammer is not designed to hammer nails, as claimed earlier, it is designed to hit things; a nail hammer is designed to hit nails, a toffee hammer to hit toffee, a warhammer to hit anyone who irritates you. In practice, a role can be considered to be a negative definition; if there is a continuant and an end-directed activity that the continuant can be involved in, and this involvement is known not to fulfil the definition of either function, then we have a role.

In this paper, I have considered OBI and found that the distinction between role and function is hard to apply; this is not true for all ontologies. For example, consider the Gene Ontology. In many cases, the homology will be considered as a standard part of the operating procedure [8] in determining the function of a gene product; regardless, the evidence codes would allow us to make the distinction. We can conclude, therefore, that when the Gene Ontology is used to annotate a protein, this describes a biological function rather than a role.

### Life is hard

So far, I have considered a set of examples and how the definitions might be applied, including examples from OBI which have not been preselected. However, categorising life is hard; here, I consider some examples which present difficulties for the definitions I have given and the implications of these examples. In the first example, which I term a* drop out* species, consider a human walking on their hands. Earlier (see "Roles"), I have suggested that this should be considered a role. Most of the primates do, however, walk on their hands. However, given that the homologous structure of closely related species use the structure for the same purpose, the definition of function (Def: 3) would appear to apply. It is for this reason that the definition specifies that (most) individuals of the same species must also demonstrate this behaviour (This definition differs slightly from that given at Bio-Ontologies 2009). In short, in the absence of most individuals in a species using a structure in a specific process, we should not use consider this structure to have a function.

The second example, I term a* drop in* species. Again, using a human example, I use my larynx for vocalisation and talking. Most primates, likewise, vocalise with their larynx; therefore, according to the given definition, this is a function of the larynx. However, speech is considered unique to humans, and therefore, their larynx; given that homologous structures in closely related organisms do not bear this realizable entity, which is part of the definition for biological function (Def: 3), I am forced to conclude that this is a role of the larynx and not a function.

In short, while sharing a realizable entity within a species is NOT sufficient to allow the conclusion that this entity IS a function, NOT sharing a realizable entity within a species is sufficient to conclude that this entity is NOT a function.

One solution to this difficulty is to state that where most individuals in a single species use a structure within a given process, this alone is sufficient to conclude that the structure has a function. Simply, most humans talk with their larynx, therefore this would be a function. I counter this, however, with the example that most humans use their fingers to operate their mobile phones, so we would be forced to conclude that this would also be a function. As this seems opposed to normal biological intuition and usage, I conclude, the presence of most individuals in a species using a structure in a specific process, is* not* sufficient to conclude that this structure has a function.

It is also possible that this difficulty could be resolved with greater knowledge or changes in biology. Def: 3 does not require species be extant; if a close, but extinct, relative of humans were shown to speak with their larynx, or if humans speciated while maintaining their speech, again, I would conclude that this represented a function.

While human speciation seems unlikely, it is much more relevant to other taxa. Bacteria, in particular, evolve rapidly. There are many genes and proteins in bacteria which are unique to a species, family or lineage [9]. In this case, the requirement for closely-related species seems to rule out the presence of a function. Again, this seems opposed to normal biological intuition and usage. I would counter this with two arguments. First, unlike primates, our knowledge of the extant bacterial species is very limited. The lack of knowledge of another speaking primate species is good evidence that no such species exists; the lack of knowledge of a close relative for a given bacterial species is not. Second, any definition which relies on a notion of a species is only as good as the definition of species; for bacteria, there is considerable debate about the utility of a species classification [10]; my definition of function will need to evolve along with our understanding of bacterial ecology and gene flow; it may be necessary, as has been suggested with definitions for bacterial species [10], to have different definitions of BiologicalFunction tailored to different parts of the taxonomy.

The evolution of a definition of role and function for proteins is difficult. At the level of the protein, I side with Dumontier [7], who suggests that the role/function distinction may be redundant; broadly, proteins can do anything their structure allows, and only do things their structure allows. The definitions given in this paper have a consistent interpretation at the level of the protein; this avoids the necessity of deciding at which level of granularity to stop making the role/function distinction. We can make the distinction at all levels if we choose, but we are not forced to do so, at those levels of granularity where it is not useful. It is important to note that arguing against a role/function distinction for proteins is not to dismiss the experience of biologists in the analysis of function assignment for genes. In this sense, the word "function" is being used to describe an association between a protein and a process that a protein molecule may be involved in; in short, the word "function", in this case, can be considered to be a synonym for "realizable entity".

## Conclusions

Here, I have taken an evolutionary approach to function and role by considering examples and using this to derive definitions which are as consistent as possible with current use within biomedical sciences. These definitions have been encoded in an axiomatisation which should enable the use of these definitions in a machine-interpretable way.

The applicability of these definitions is a key advantage; the current distinction being made between function and role is a hard one to understand and apply. My definition distinguishes between the two based on the nature of the relationship to the independent continuant in which they inhere. I suggest that it is very hard to make the distinction at the class level; my study of OBI shows that very few of the functions and roles clearly fall into one category or another. For an individual continuant bearing a realizable entity, this distinction appears to be much more straightforward.

I also provide a definition of biological function, something that is currently lacking in BFO. I have paid close attention to current biological usage; the definition is close to the process used to determine function. Moreover, it is highly applicable; all parts of the definition are measurable.

The desire for an applicable and measurable definition is also the reason that I have avoided a definition based on the outcome of selective pressure; this is hard to test in most circumstances, requiring expensive evolutionary studies, and impossible for extinct species. Selective pressure can also be transient. Consider industrial melanism [11]; should melanic coloring be considered to gain its function during periods of pollution and lose it in post-industrial periods? By way of analogy, should a spanner measured in inches be considered to lose its function following metrication? Serendipitously, it also avoids difficult questions about artificial selection; we can state clearly that cows do not have a function of producing beef, though this is the outcome of selection.

Importantly, my definition of biological function works across multiple levels of granularity: from organisms and organism parts through to genes and molecules; this is not true of previous definitions [4], which cover only anatomy. It is, however, not clear how useful the role/function distinction is for proteins and genes, as discussed earlier (see "Life is hard"); It is for this reason that I have used homology rather than orthology as the basis for the definition, as the latter is limited to the genetic scale, where the distinction is least useful.

Finally, my definitions also do not allow distinctions that may often be made between different types of function. For example, most biologists would consider motion the most important function of muscle, while heat production a byproduct; or, for a more pathological example after Hoehndorf et al. [12], most biologists would consider blood circulation to be a function of the heart, but "making loud thumping noises" not to be. This is a concern which could be best addressed by incorporating a degree of social ascription into the categorisation of realizable entities within biology; although it is outside the scope of this paper, this would provide a valuable and useful addition to the current ontological practice. In summary, I believe that the definitions and axiomatisation given in this paper make a significant contribution to the use of role and function in biomedical ontologies. They should enable a consistent use of these classes, because they consider current usage of the terms and the applicability of these definitions. I seek not to change current use but to formalize it.

## Competing interests

The author has no competing interests.
